# Types of Cognitive Appraisal and Undertaken Coping Strategies during Sport Competitions

**DOI:** 10.3390/ijerph17186522

**Published:** 2020-09-08

**Authors:** Kamila Litwic-Kaminska

**Affiliations:** Faculty of Psychology, Kazimierz Wielki University, Leopold Staff Str. 1, 85-867 Bydgoszcz, Poland; k.litwic@ukw.edu.pl

**Keywords:** stress, primary appraisal, coping, athletes

## Abstract

The main aim of the research was to distinguish different types of sport competition appraisals and verify if athletes’ interpretation of a stressful situation changed their choice of coping methods. Athletes change their perception during competitions; thus, we assumed that configuration of different ways of interpreting stressful events is more important for coping than one particular appraisal. In total, 193 athletes filled out The Stress Appraisal Questionnaire and The Sport Stress-Coping Strategies Questionnaire to describe their stress appraisals and undertaken coping strategies during a remembered competition that took place within a month before the study. The athletes most often appraised stressful competitions as a challenge. They preferred the coping strategy of being determined to accomplish the established goal. The athletes hardly applied techniques that constituted the basis of mental training. The cluster analysis of the competitors determined three types of sport competition appraisals: positive, negative, and active. An ANOVA with post hoc comparisons showed that participants who revealed positive appraisals undertook the highest number of actions aimed at reaching goals and least frequently sought support. Athletes should be taught not only specific strategies for coping with stress, but also more frequent use of positive judgments of sports competitions.

## 1. Introduction

Physical activity is perceived as one of the most important aspects of lifestyle that has a positive effect on both physical and psychical wellbeing of a person. Indeed, an average person reaps benefits from regular yet moderate sport activity. However, professionals train in order to achieve a high level of mastery and not necessarily to remain in optimal health. Competitors obtain results of extreme character, which results in every sport achievement being tantamount to the experience of stress. People involved in sport struggle with many emotional difficulties regarding rivalry, training, time pressure, or the life beyond sport, which considerably influence the manner of perception and coping with stressful situations. On the other hand, providing gradual loads through sports training shapes an athlete’s resistance to stress. In addition to sports preparation, mental skills training can be used to shape emotional control skills through relaxation, imagination, or simulation training. This explanation is supported by Dugdale, Eklund, and Gordon [1], who showed that athletes who rated their coping as being more automatic (as a consequence of training) saw this coping as more effective. Successful training (both physical and mental) should lead to a situation in which loads that signify exhaustion among ordinary people still remain in the resistance stage for athletes. Effective coping with stress is, therefore, an important skill which, apart from the increase in sport advancement, enables maintaining a high quality of life or even mental health [2,3,4].

One of the most often referred concepts of stress in relation to athletes [5] is the transactional theory of stress by Lazarus and Folkman [6]. In this concept, stress is described as a dynamic relationship between human and environment in which both sides mutually influence each other. According to this concept, the primary appraisal of a situation is the first step in the process of stress, which determines its further development. Cognitive appraisal has an important place in the process of coping with stress in professional sports. It enables a person to decide whether a situation is perceived as stressful or not. When athletes do not perceive a situation as stressful or as positive, they do not need to undertake steps aimed at coping with it. Contrary, if a situation threatens one’s wellbeing and an event is appraised as stressful—as a threat, loss, or challenge—the person starts undertaking actions aimed at coping with it. Earlier studies showed that the way athletes interpret stressful events is related to the level of their perceived intensity of the stress, their experienced emotions, their coping styles, and their level of performance [7,8,9]. Regarding stressful situations as losses may be related to lower levels of self-confidence or feelings of hopelessness. Perceiving situations as threats may lead to doubt in their own skills or to anxiety about possible loss. When athletes see a situation as a challenge, they may feel confident about being able to take action, control the situation and, thus, develop a strong motivation to prepare well for the competition [10]. The secondary evaluation is connected with the estimation of capabilities when taking up certain actions in order to remove the cause of stress or at least some methods of soothing its ravages or, in case of challenge, leading to the achievement of available profits. Both cognitive processes—primary and secondary evaluation—run simultaneously and are associated.

Just like many other psychological terms, the notion of stress coping is highly ambiguous. Coping with stress may be considered as a process, strategy, or style [11]. The term process concerns the complex and dynamic activity taken up in face of a stressor, which lasts for the time of its operation (often very long, e.g., chronic illness) and is changeable under the influence of the stressful situation’s development. Such an approach was represented by Lazarus and Folkman [6]. It should be emphasized that the course of the stress process depends on the situational context. Nicholls, Levy, Grice, and Polman [12] found in their study of runners that athletes indicated different stressors and used diverse coping strategies during training and competition days. Therefore, in this paper, we use the term “strategy” as an element of the coping process. Under this term, there are specific actions and reactions that people undertake in particularly stressful situations. The term strategy is used in contrast with the concept of coping styles, which describe a relatively permanent, habitual behavior of people in stressful situations [11].

Lazarus and Folkman’s [6] work discussed problem- and emotion-focused coping. A concept of coping widely used within sport [13] is the theory developed by Roth and Cohen [14], in which they distinguished two categories of coping strategies in a stressful situation: approach and avoidance. Approach coping includes taking direct action, increasing effort, and planning. Avoidance coping involves behavioral and psychological efforts to release oneself from a stressful situation. Another popular sport-related categorization involves three types of coping: problem-focused, emotion-focused, and avoidance coping [5]. Regardless of the coping classifications, generally, task-oriented or problem-focused coping is considered to be the most commonly used and the most functional, i.e., being the most effective dealing with problems [15,16,17,18]. The results of the published research conducted on sportsmen related to the strategies of coping allow for the assumption that sportsmen engaged in various disciplines show certain specified types of strategies that stem from experiencing intensive stress during competition [19,20]. For example, during competitions, especially in motor performance, the avoidance method can be more effective [19,21], although this coping-style approach can be time-consuming and distractive. When using the avoidance coping style, contestants do not pay attention to the stressful stimuli, which enables them to concentrate on the action and obtain positive results. The effectiveness of coping with stress depends not only on the use of a coping style that the competitor recognizes as beneficial, but also applying various strategies dependent on individual differences as a function of their perception of the intensity of the stress [20]. In turn, according to the goodness-of-fit hypothesis [17], the effectiveness of different coping strategies varies depending on how the athlete evaluates the stressing situation. 

According to the transactional theory of stress [6], there are dynamic changes in the perception of a situation and the resultant coping choices when the processual nature of stress is taken into account. However, most authors of previous research on coping only dealt with the associations between particular kinds of appraisals (threat, loss, or challenge) and strategies used for coping with perceived stress. Some of them paid additional attention to the relationship between appraisal and coping and found that this can be mediated by one’s emotional reaction to stress [8,22]. Therefore, it was assumed that athletes change their perception during competitions; thus, the configuration of different ways of interpreting stressful events is important. The main aim of this study was to distinguish the types of appraisal of sport competitions and to verify how athletes’ interpretations of stressful situations changed their choice of coping methods. I hypothesized the configuration of an athlete’s evaluations of stressful competition is related to the choice of specific coping strategies. 

## 2. Materials and Methods

### 2.1. Participants

The presented results were developed on the basis of data collected from 193 athletes (95 women and 97 men) from the following summer Olympic disciplines: shooting, handball, rowing, combat sports (judo and taekwondo), volleyball, and football. They were all between 17 and 38 years old, but the majority of them were 25 years old or younger (90%). Using the categorization of elite athletes given by Swan et al. [23], the level of the competitors was marked according to the type of competition they regularly took part in (international/national competitive level) among individual athletes and according to professional leagues in team disciplines. The largest number of individual participants took part in nationwide competitions (*n* = 60). Other individual athletes participated in World (*n* = 19) and European (*n* = 15) championships or took part in junior provincial competitions (*n* = 3). Among team disciplines, 21 surveyed team athletes were playing in the top (*n* = 21), first (*n* = 19), second and third (each *n* = 11), fourth (*n* = 16), and junior (*n* = 18) leagues. Neither sample size calculation nor power analysis was performed a priori. A detailed characteristic of participants is presented in the Table 1.

### 2.2. Procedure

Studies were conducted during the training camps and in training sessions in various clubs throughout Poland among adult athletes who voluntarily participated. Each participant filled out an informed consent form that was accompanied by written study information, a set of psychological questionnaires, and a survey of sociodemographic information (gender, age, and sport-related information: type of discipline, practice time, and sport level).

The criteria for choosing the examined disciplines were determined on the basis of specific situations in which the contestants found themselves during competitions and included a combination of the following aspects: individual/team performance, direct/indirect rivalry, and the degree of physical contact between players. Gender and age distributions were controlled for in particular disciplines to ensure an equal balance between the number of women and men and a balanced spread of ages. We also used the principle to collect and examine a maximum of five participants who were practicing with any given coach to exclude any potential impact from the respondents having a common environment. 

For ethical and organizational reasons, the study did not take place during any competitions; rather, athletes referred to a remembered situation that took place within a month before the study. At the beginning of the questionnaires was a place where the participants were asked to write a brief description of a stressing situation during their particular remembered competition or match. 

All subjects gave their informed consent for inclusion before they participated in the study. The study was conducted in accordance with the Declaration of Helsinki. However, studies were not confirmed by written consent of the ethics committee. According to Polish legislation, written approval from the ethics committee in studies using retrospective questionnaires with minimal ethical risk (i.e., that do not violate the physical integrity of participants or do not have a temporary, fluctuating, or permanent impairment in or disturbance of the functioning of the mind or brain, or where the research group does not include particularly vulnerable individuals and the data collected in the study are anonymized) was not necessary before the start of the study.

### 2.3. Measures

On the provided self-report questionnaires, the athletes described particular competitions they perceived as stressful and evaluated their situational appraisals and coping strategies. 

The Stress Appraisal Questionnaire (SAQ), version A to assess situational stress appraisal [24] consists of a list of 35 adjectives used to describe a stressful situation where 23 of them are diagnostic items and the rest have a buffer function. Version A allows the user to measure situational appraisals that are connected to a particular situation (“This situation was for me…”, e.g., terrifying). Responses are given on a four-point scale (0–3 points); the tool allows users to distinguish four types of stressful situation appraisals: threat (nine items), harm/loss (four items), challenge-activity (five items), and challenge-passivity (five items). Reliability coefficients for the subscales were acceptable or good, between 0.71 and 0.90 [24]. The questionnaire was developed for a group of patients after myocardial infarctions, and it was not previously used with a group of athletes, which limits potential comparisons. Therefore, a confirmatory factor analysis (CFA) was performed using AMOS v25 (IBM SPSS, Chicago, IL, USA) [25] to ensure the questionnaire was appropriate for the population of athletes. The maximum-likelihood procedure was used. The following values for the adjustment indices were obtained: the χ^2^ value/degrees of freedom (CMIN/DF) was 1.603, the comparative fix index (CFI) was 0.944, the root-mean-square error of approximation (RMSEA) was 0.056, the Tucker–Lewis index (TLI) was 0.934, and the residual standardized root mean square (SRMR) was 0.064. The goodness-of-fit indexes met the established criteria [26]: CMIN/DF < 3, CFI > 0.90, RMSEA < 0.08, TLI < 0.95, and SRMR > 0.06. Similar results in Cronbach’s alphas obtained in this study (between 0.68 and 0.89) and the results of CFA suggest that this tool works well in a group of athletes who experience strong or prolonged stress.

The Sport Stress-Coping Strategies Questionnaire (SR3S, 27) was constructed for this study due to the lack of tools for measuring stress-coping strategies with regard to a specific competitive situation available in the Polish language. The currently applied tools primarily allow examining general life situations, but do not include specific situations involving an athlete or stress-coping techniques, which are recommended for athletes in mental training. The questionnaire includes 22 statements (with a five-point scale) about possible actions and reactions undertaken in stressful situations, grouped into four scales: setting the goal/victory, seeking support, applying mental techniques (e.g., relaxation), and planning/focusing on the activity. Cronbach’s alphas for particular scales were between 0.75 and 0.83 [27]. The SR3S has not yet been subjected to a standardization procedure. It is necessary to conduct further research with it in order to make comparisons.

### 2.4. Data Analysis

Statistical analysis was performed in Statistica v13 (Stat Soft, Tulsa, OK, USA). To illustrate the types of appraisals of stressful sport competitions and to exemplify the strategies used by athletes, descriptive statistics (mean, standard deviation, skewness, kurtosis) were performed. In order to characterize the examined contestants despite a different number of statements in particular subscales of the Stress Appraisal Questionnaire, within each variable, the weighted arithmetic means were divided by the number of statements included in a given factor. The cluster analysis of the competitors was used to determine the athletes’ types of appraisal of their sport competitions. At first, the number of possible clusters was determined on the basis of the agglomerative hierarchical clustering. Then, all data were divided into clusters through k-means clustering. After that, nonparametric tests were employed to verify the significance of the differences between the obtained mean values for each type of appraisal in each cluster (due to the lack of a normal distribution). The Kruskal–Wallis ANOVA test was used to analyze the divergences between clusters. The differentiation within clusters was validated with the Wilcoxon test. At the end, to verify if the athletes’ interpretation type for a given stressful situation changed their choice of coping methods, a one-way ANOVA with post hoc comparisons (Fisher’s Least Significant Difference test–LSD) was performed. The Eta-squared (η^2^) estimator was used to measure the effect size for significant comparisons in ANOVA. Additionally, a *t*-Student test of independent samples was carried out in order to verify the significance of the differences between the mean values of each coping strategy in the examined subgroups. Statistical significance was defined for *p*-values lower than 0.05.

## 3. Results

### 3.1. Appraisal of Stress and Undertaken Coping Strategies during Sport Competitions

With regard to the dominating method of cognitive appraisal, the majority of athletes perceived competitions as a challenge that led to increased activity (60% of participants). The remaining athletes appraised competitions as a challenge that did not trigger action (18%), as a loss (16%), or as a threat (5%). Within the types of stress-coping strategies during competitions, athletes most often aimed to achieve a certain goal or victory (mean (M) = 3.89, SD = 0.83). The least frequent strategies employed during stressful competitions were those related to seeking support (M = 2.76, SD = 0.92) and using mental techniques (M = 2.62, SD = 1.11). Particular means and SDs are shown in Table 2.

### 3.2. The Types of Stress Appraisal

**Figure 1:**
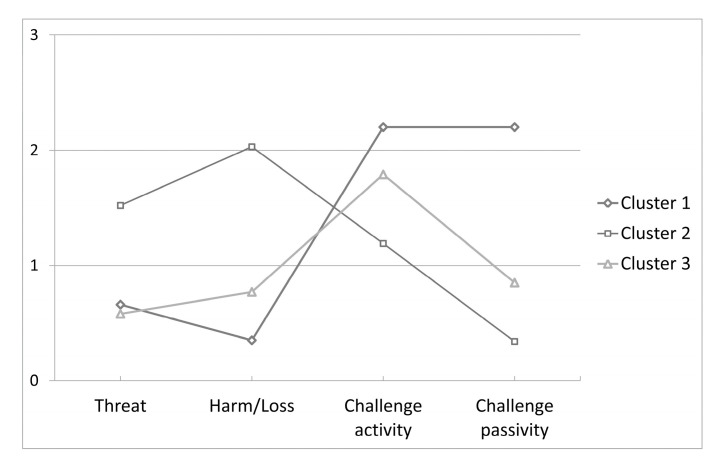
Mean values for the clusters of competitors appraising competitions similarly.

After the cluster analyses, it was possible to acknowledge that the examined athletes were characterized by three ways of appraising competitions (Figure 1). The most important criterion determining the assignment to clusters was the assessment of challenge-passivity (F_2, 190_ = 372.61, *p* < 0.001, η^2^ = 3.92) or harm/loss (F_2, 190_ = 163.33, *p* < 0.001, η^2^ = 1.72). The Kruskal–Wallis ANOVA and Wilcoxon tests showed divergences between and within clusters. Apart from the differences between groups 1 and 3 in threat appraisal, all mean values of the cluster groups significantly varied with respect to each other (*p* < 0.05). The first and biggest group of competitors (cluster 1, *n* = 87, around 45%) most intensively appraised the situation as a challenge connected to both activity and lack of activity (M = 2.20, SD = 0.39 and 0.41, respectively). Significantly less frequent was the appraisal as a threat (M = 0.66, SD = 0.45), and the least frequent was appraisal as harm/loss (M = 0.35, SD = 0.37). In the second group (*n* = 41, around 21%), most of the athletes appraised stress as harm/loss (M = 1.52, SD = 0.60) or as a threat (M = 2.03, SD = 0.59). Least frequent was the appraisal as a challenge (activity M = 1.19, SD = 0.56; passivity M = 0.34, SD = 0.35). In the last group (*n* = 65, around 34%), the appraisal of the stressful situation as a challenge connected with activity (M = 1.79, SD = 0.57) dominated (threat M = 0.58, SD = 0.49; harm/loss M = 0.77, SD = 0.56; challenge-passivity M = 0.85, SD = 0.43). On the basis of these data, three types of appraisal used by athletes were recognized and were, respective to the clusters, positive, negative, and active.

### 3.3. The Types of Appraisal against the Undertaken Stress-Coping Strategies

The ANOVA test calculations suggest that there were significant differences among the groups presenting certain types of situation appraisals with regard to their chosen undertaken coping strategies during competitions (Table 3). The biggest number of differences appeared within those seeking support (F_3, 190_ = 4.01, *p* = 0.020, η^2^ = 0.041), but the types differed also in setting the goal (F_3, 190_ = 4.86, *p* = 0.009, η^2^ = 0.049) and planning/action (F_3, 190_ = 3.79, *p* = 0.024, η^2^ = 0.038) strategies. The active group least frequently undertook seeking support (*p* < 0.05). In turn, contestants characterized by positive types of appraisal most often applied strategies aimed at reaching the goal (*p* = 0.003).

## 4. Discussion

The results of this research indicate that, during sport competitions, athletes most often apply positive stress appraisals (seeing them as a challenge). This finding conforms with the results of prior research (e.g., [8,9]) where athletes appraised stress more as a challenge than as a threat. In prior scientific reports, other authors indicated the existence of a relationship among appraising stressful situations as a challenge, increasing faith in one’s capacity to act, finding greater motivation to work hard, increasing one’s sense of control over a situation [9], and obtaining better sport results [7]. In the light of these findings, my study’s outcomes suggest that athletes have a good attitude toward competitions. 

Additionally, the cluster analysis allowed distinguishing the types of athletes who perceived their situations in a certain way. Most of the examined athletes (79%) presented a positive appraisal of the sports competition situation. These are the athletes who appraised challenges in the types specified in this study as positive and active. There were also some athletes who primarily perceived stress during the competition in a negative way, which indicates a possible direction for future research (e.g., performing discriminatory or regression analysis) to find the individual differences or situational determinants between athletes belonging to positive and negative groups. This could help to determine the likely causes of less favorable, negative evaluations, which, in turn, would allow suggesting the methods that work best to sports psychologists or coaches.

The examined competitors preferred task-oriented coping strategies, as well as rarely applied techniques, which form the basis of the mental training carried out by sport psychologists. Considering the most often applied strategies connected with concentrating on the goal and victory and with planning and concentrating on activity, one could enquire what incentive contemporary athletes follow. Concentrating on winning as a form of coping with stress may be a reflection of such a tendency in sports where athletes seek external rewards, whether material (medals, cups, or financial gratification) or immaterial (e.g., social prestige). Nevertheless, it should be emphasized that inappropriate forms of external motivation may decrease or eliminate an athlete’s internal motivation [28] or increase one’s cognitive anxiety [29]; thus, the way coaches or club managers offer praise or motivate their athletes is very important. Furthermore, the reason that mental techniques are rarely used as coping strategies is uncertain. It could be the lack of athletes’ willingness, the necessities involved in their application, the athletes’ inability to use them, or simply their lack of knowledge about them. Additional interviews with the participants indicated the two latter options were most likely. This may be due to the still insufficient availability of sports psychologists or Poland’s unreadiness to use their services. Prior investigations suggested that mental training is an effective way of improving people’s quality of functioning when they are experiencing strong emotions (e.g., [30]). Therefore, it seems worthwhile to carry out a deeper study into the usage of such techniques and the demands Polish athletes have regarding their usage. 

Referring to the main purpose of this study, certain types of situation appraisals influence the frequency of an athlete using certain strategies in sports competitions. All athletes, regardless of their perception of stress, usually undertake task coping. It can be assumed that, whether or not they are afraid of the stressful situation, they are mobilized and, during competitions, they take actions they consider to be the most effective for coping with stress. However, previous findings showed that dealing with stress by focusing on the task and trying to solve the problem is not always the most effective way [19]. According to the transactional stress theory [6] and the coping flexibility hypothesis [31], it can be assumed that being more flexible about choosing how to cope is associated with more adaptive outcomes. Therefore, athletes’ repertoire of coping skills should include a whole range of possibilities in order for them to be more effective in various situations. In our study, athletes who were classified as positive undertook the largest number of activities in order to cope with stress.

Furthermore, athletes presenting negative types of appraisal, most often of all types, used strategies involving seeking social support. Perhaps this was how they coped with their fear or uncertainty before the competitions started. Pons, Viladrich, and Ramis [32] showed that coping, including efforts to regulate internal stress responses, is connected with anxiety and seeking social support.

Our results are consistent with the studies on the big three elements of coping in sports—cognitive appraisal, coping strategies, and emotions [32]. Assuming a mediating role of emotions between appraisals and coping [8], it can be considered that different appraisals lead to different emotions and are coped with using different strategies [33]. Therefore, athletes should be taught not only specific strategies for coping with stress, but also more frequent use of positive judgments of sports competitions (or particular situations during these events). This can be achieved through training their positive internal dialogue, for example, by thinking “I can do it” or “I am ready”, while reducing the number of negative thoughts associated with feeling a threat or loss (such as thinking about avoiding an error). Other methods may be visualization (imagery), stimulating positive emotions in stressful situations, using arousal-inducing techniques, goal reengagement, or self-presentation manipulation [34,35,36]. Furthermore, increasing athletes’ self-awareness can lead them to use seemingly negative threat appraisals as alarms against potential problems and help prevent them, or it can lead to faster coping with adversity [13].

This paper contains certain limitations that may provide guidance for carrying out future research in this field. First, the size and heterogeneity of our research group (athletes of different disciplines) limit the ability to generalize our research findings. No power analysis was performed to evaluate the sample size. Despite the fact that the age distribution in all disciplines was particularly focused on, there were some discrepancies arising from the specifics involved in training each type of sport. Because athletes are often unable to continue shooting and combat sports as they grow older, since they usually only train until they have to study, work, or start a family, it was difficult to reach the most experienced (and age-matched) competitors. Another limitation of our research is that the examined athletes participated in competitions at objectively different levels (international, national, or local) which may have influenced the way they coped. On the other hand, athletes competing in high-level competitions may have more experience and perceive stressing situations differently than less experienced participants [37]. This was also confirmed by additional analysis performed related to this study. Correlation analysis indicated that the older and the more experienced athlete playing at a higher competitive level more often undertake challenge appraisal and strategies connected to concentrating on the goal and victory or to planning and concentrating on an activity. Moreover, older and more experienced athletes more often use mental techniques. Thus, on the contrary, a heterogeneous population can be a positive point. In this perspective, the data could be valid for several athletes in different sports. It would be necessary to find differences between particular disciplines. The relatively small sample size makes this difficult. This needs to be clarified in further research.

Another issue concerns the limitations of self-report questionnaires. Stone et al. [38] showed that real-time measurement differs from retrospective coping assessments. The participants more likely underreported cognitive coping and overreported behavior coping retrospectively. Due to the difficulty of writing anything down during sports competition in most sport disciplines, the think-aloud (TA) method is often proposed [18,39] as a form of ecological momentary assessment. Indeed, TA has the advantage of eliminating errors caused by retrospective recall, but this method is not free from other limitations. TA appears to be appropriate for measuring acute stress, but will not detect longer-lasting, more complex aspects of stress or coping methods that require time for retrospection, e.g., a positive reframing of the meaning of the event [39,40]. Nicholls et al. [39] noticed that TA appears more suitable for measuring cognitive strategies for coping than behavioral strategies. Although TA represents a useful idea to collect stress and coping data, it is difficult to implement it in many sports. It would be a feasible measurement method in sports like golf, sailing, or archery, but awkward to execute in more aerobically demanding or team sports. Structured interviews are another frequently used alternative to self-report questionnaires [13], but these are very time-consuming methods and they require the trust of the respondents. Thus, in this study, we chose questionnaires, which seem to be one of the most easily accessible and beneficial research methods when comparing different disciplines.

## 5. Conclusions

During competitions, athletes most often apply positive types of stress appraisal (as a challenge), as well as problem-focused coping strategies (aimed to achieve a certain goal or victory and connected to planning or focus on an activity). Moreover, people who apply positive appraisals undertake the largest number of activities in order to cope with stress; thus, they may be considered the most flexible in coping. The results from this study extend the literature on stress and coping by introducing a new view of stress appraisal as a configuration of different ways of interpreting stressful events, as opposed to treating particular appraisals as separate aspects. From a practical perspective, athletes should be taught not only specific strategies for coping with stress, but also more frequent use of positive judgments of sports competitions. Athletes need to be able to cope with stressors associated with competition, not only to achieve the best results, but also to enjoy their sports experience.

## Figures and Tables

**Table 1:** Participants characteristics with regard to sport discipline.

Sport Discipline	*N*	Gender (*n*)		Age	
		Female	Male	M (SD)	Range
Combat sports	32	16	16	19.97 (3.21)	17–30
Football	31	16	15	21.42 (5.14)	17–38
Handball	33	16	17	20.30 (3.09)	17–27
Rowing	32	15	17	20.88 (3.26)	17–29
Shooting	33	18	15	18.21 (1.58)	17–22
Volleyball	32	15	17	20.94 (3.26)	17–30
Total	193	96	97	20.27 (3.51)	17–38

**Table 2:** Appraisal of stress and undertaken coping strategies during sport competitions.

Variable	M (SD)	Range	Skewness	Kurtosis
Cognitive appraisal				
Threat	0.82 (0.62)	0–2.78	1.01	0.83
Harm/Loss	0.85 (0.81)	0–3	0.91	0.07
Challenge-activity	1.85 (0.63)	0.2–3	−0.42	−0.14
Challenge-passivity	1.35 (0.89)	0–3	0.05	−1.20
Coping strategies				
Setting on the goal/victory	3.89 (0.83)	2–5	−0.54	−0.68
Seeking support	2.76 (0.92)	1–5	0.15	−0.63
Applying mental techniques	2.62 (1.11)	1–5	0.30	−0.83
Planning/focus on activity	3.51 (0.75)	1–5	−0.33	0.22

**Table 3:** Differences in the undertaken coping strategies with regard to the type of cognitive appraisal of competition, using ANOVA with post hoc comparisons (Fisher’s LSD test).

Strategies	F	*p*	η^2^	Type of Appraisal	M	*p*-Values		
						P	N	A
Setting on the goal	**4.86**	**0.009**	**0.049**	P	4.07		**0.003**	0.073
				N	3.60	**0.003**		0.164
				A	3.83	0.073	0.164	
Seeking support	**4.01**	**0.020**	**0.041**	P	2.84		0.421	**0.027**
				N	2.98	0.421		**0.010**
				A	2.51	**0.027**	**0.010**	
Using techniques	1.53	0.220	0.016	P	2.74	**------------**		
				N	2.68			
				A	2.43			
Planning/action	**3.79**	**0.024**	**0.038**	P	3.67		0.081	**0.010**
				N	3.43	0.081		0.628
				A	3.35	**0.010**	0.628	

P: positive; N: negative; A: active type of cognitive appraisal; *df* = 190; the significant (*p* < 0.05) results are shown in bold.
